# Nutraceuticals for oral health: scientific rationale and clinical potential in endodontics

**DOI:** 10.2340/aos.v85.45029

**Published:** 2026-01-02

**Authors:** Romulo de Oliveria Sales-Junior, Renan Dal-Fabbro, Luciano Tavares Ângelo Cintra, João Eduardo Gomes-Filho

**Affiliations:** a Department of Preventive and Restorative Dentistry. São Paulo State University (Unesp), School of Dentistry, Araçatuba, São Paulo, Brasil; b Department of Cariology, Restorative Sciences, and Endodontics, University of Michigan School of Dentistry, Ann Arbor, Michigan, USA

**Keywords:** Nutraceuticals, endodontics, systemic health, oral health

Dear Editor,

The scientific understanding of the link between nutrition and disease modulation has grown significantly since the early discoveries of micronutrient deficiencies established the vital role of diet in health and disease management [1]. The concept of nutraceuticals (bioactive substances derived from foods that provide health benefits beyond basic nutrition) has gained prominence in both research and clinical practice [2]. Unlike conventional pharmaceuticals, nutraceuticals are not intended to replace standard therapies; rather, they are increasingly recognized for their potential as complementary agents in disease prevention and treatment [1]. Studies have begun to elucidate the mechanisms by which various nutraceutical compounds exert anti-inflammatory, antioxidant, and immunomodulatory effects, with promising results in chronic, multifactorial conditions such as metabolic syndrome, cardiovascular disease, and certain inflammatory disorders [1, 2].

Several medical specialties have already integrated nutraceuticals into their therapeutic protocols. In dentistry, they have been explored across several subfields for their potential to modulate inflammatory pathways, oxidative stress, and the oral microbiome [3, 4]. The strongest evidence to date comes from periodontology, where bioactive compounds show promise for controlling inflammation and supporting tissue repair [3, 4]. In cariology, nutraceuticals may help prevent dental caries by inhibiting cariogenic pathogens and maintaining microbial balance, while in orthodontics, they may reduce plaque accumulation and gingival inflammation [4]. In implantology, probiotics, polyphenols, and vitamins have been linked to improved osseointegration [4]. In oral medicine, they may help preserve mucosal integrity and prevent common oral diseases [4]. However, despite these advances, the role of nutraceuticals in endodontics remains largely unexplored. One emerging concept is that nutraceuticals could restore the balance of metabolic cofactors and essential nutrients in affected tissues, potentially attenuating the inflammatory cascade associated with infection and tissue injury, as in endodontic disease [3, 4]. To date, there is only limited experimental evidence and a lack of clinical trials evaluating whether adjunctive nutraceuticals can support periapical healing or help control the local and systemic inflammation associated with endodontic infections. This knowledge gap is noteworthy and demands attention, given the interplay between endodontic disease and systemic health.

It is essential to consider the interplay between apical periodontitis (AP) and the host’s systemic condition, particularly their immune and nutritional status, when exploring new adjunctive therapies. AP is not merely a local pathology; increasing evidence suggests that an individual’s immune competence influences the initiation and progression of AP, as well as treatment response and healing outcomes [5, 6]. An exaggerated local immune-inflammatory reaction in a periapical lesion can also have systemic spillover effects, potentially triggering or exacerbating systemic conditions in predisposed individuals, although the exact biological pathways remain incompletely understood [5–7]. This host-pathogen interaction is significant, especially given the high global prevalence of AP, which affects nearly 50% of adults worldwide [7]. Moreover, several patient factors, including chronic systemic diseases, age, smoking habits, and particularly nutritional status, have been shown to influence periapical healing after endodontic treatment [5–8]. In light of these observations, it is reasonable to ask whether improving a patient’s nutritional and immune status through nutraceutical supplementation could enhance the healing of endodontic lesions and possibly mitigate any related systemic impact.

Emerging evidence suggests that nutraceutical supplementation could benefit endodontic outcomes by directly modulating periapical inflammation and supporting bone repair [3–8]. It has been demonstrated, for instance, that inadequate levels of essential nutrients can impair tissue repair, immune regulation, and bone metabolism, all critical factors for the resolution of periapical lesions [6]. Consistent with this, nutritional imbalance has been identified as a significant systemic determinant that can adversely affect healing outcomes in endodontic therapy [5].

Various bioactive compounds are being explored for their therapeutic effects in this context. Examples include resveratrol, quercetin, curcumin, omega-3 fatty acids, propolis, melatonin, and probiotics, as well as plant extracts like açaí berry and Yerba Mate (*Ilex paraguariensis*) [8–16]. Collectively, these nutraceuticals exhibit notable antioxidant, anti-inflammatory, and immunomodulatory properties. They can reduce oxidative stress, neutralize free radicals, and downregulate pro-inflammatory cytokines while stimulating host defense mechanisms [8–16]. Many of them also influence bone metabolism by promoting osteoblastic activity and inhibiting osteoclastic resorption, which may contribute to the repair of periapical tissues following endodontic infection and treatment [8–16].

Ultimately, the goal of adjunctive nutraceutical therapy in endodontics is to recalibrate the host’s immune response by reducing excess pro-inflammatory mediators and tissue-degrading enzymes while enhancing anti-inflammatory factors and natural inhibitory molecules [1–3]. Importantly, this strategy is not intended to replace standard antimicrobial disinfection of the root canal system, but to complement it by strengthening the host’s defenses and modulating the inflammatory cascade [1–3]. In essence, these natural agents act as biological response modifiers, influencing molecular pathways that govern tissue breakdown, immune cell recruitment, and bone remodeling during the post-treatment healing phase. Such an adjunctive approach may open new possibilities for optimizing endodontic treatment outcomes [1–3, 8, 9].

Despite the strong scientific rationale outlined above, current evidence for the use of nutraceuticals in endodontics is largely limited to preclinical studies. We lack clinical trials evaluating nutraceutical supplementation as an adjunct to standard endodontic therapy. Given the biological plausibility and the potential for a more universal treatment approach, we believe that the endodontic research community can further investigate whether such interventions can improve periapical healing and potentially benefit patients’ systemic health. Moreover, this concept may encourage broader investigation of nutraceuticals across dental specialties, moving from robust preclinical models to well-designed translational and clinical trials. Such progress is essential for developing evidence-based guidelines for the clinical use of nutraceuticals in oral health and their integration with endodontic therapy. We hope this correspondence will stimulate interest, discussion, and further research on this promising yet underexplored topic within our field.
